# Downregulation of hTERT: An Important As_2_O_3_ Induced Mechanism of Apoptosis in Myelodysplastic Syndrome

**DOI:** 10.1371/journal.pone.0113199

**Published:** 2014-11-21

**Authors:** Weilai Xu, Yungui Wang, Hongyan Tong, Wenbin Qian, Jie Jin

**Affiliations:** Institute of Hematology, First Affiliated Hospital, College of Medicine, Zhejiang University, Hangzhou, Zhejiang, China; Georgetown University, United States of America

## Abstract

Two myelodysplastic syndrome (MDS) celllines, MUTZ-1 and SKM-1 cells, were used to study the effect of arsenic trioxide (As_2_O_3_) on hematological malignant cells. As_2_O_3_ induced this two cell lines apoptosis via activation of caspase-3/8 and cleavage of poly (ADP-ribose) polymerase (PARP), a DNA repair enzyme. As_2_O_3_ reduced NF-κB activity, which was important for inducing MUTZ-1 and SKM-1 cells apoptosis. As_2_O_3_ also inhibited the activities of hTERT in MUTZ-1 and SKM-1 cells. Moreover, the NF-κB inhibitor, pyrrolidine dithiocarbamate (PDTC), had no effect on caspase-8 activation, although PDTC did inhibit MUTZ-1 and SKM-1 cells proliferation. Incubation of MUTZ-1 cells with a caspase-8 inhibitor failed to block As_2_O_3_-induced inhibition of NF-κB activity. Our findings suggest that As_2_O_3_ may induce apoptosis in MUTZ-1 and SKM-1 cells by two independent pathways: first, by activation of caspase-3/8 and PARP; and second, by inhibition of NF-κB activity, which results in downregulation of hTERT expression. We conclude that hTERT and NF-κB are important molecular targets in As_2_O_3_-induced apoptosis.

## Introduction

Arsenic trioxide (As_2_O_3_), an inorganic compound originally isolated and used by traditional Chinese medicine as a therapeutic reagent, is an effective drug for treating acute promyelocytic leukemia (APL) [1]–[3]. APL patients who are resistant to conventional chemotherapy and all-trans retinoic acid have a high response rate to As_2_O_3_ treatment. Numerous experimental and clinical investigations have demonstrated that As_2_O_3_ induces apoptosis in cancer cell lines, and is an effective drug in the treatment of patients with hematological malignancies, including APL, acute myeloid leukemia (AML) [4], [5], multiple myeloma (MM) [6], [7], myelodysplastic syndromes (MDS) [8]–[10], and non-Hodgkin's lymphoma [11], [12].

However, the pharmacological mechanism of As_2_O_3_ in the treatment of hematological malignancy remains unclear. Previous studies have demonstrated that As_2_O_3_ induces apoptosis in malignant cells, such as NB4 cells (an APL cell line) and endometrial cancer cells [13], [14]. In addition to inducing apoptosis, As_2_O_3_ may have an antitumor effect on endometrial carcinoma cells by inhibiting hTERT mRNA transcription and telomerase activity [14]. Further studies have shown that As_2_O_3_ inhibits NF-κB activity [15], [16], which is a known transcriptional regulator of hTERT expression. hTERT regulates cell survival and apoptosis in response to external stress and has a critical role in the development and function of multiple tissues and organs [17], [18]. In the hTERT promoter there are several binding motifs for various transcription factors, including NF-κB, Myc/Mad (E box), Sp1, and estrogen. NF-κB p65 regulates telomerase via nuclear translocation of the hTERT protein. Sinha-Datta et al suggested that hTERT was transcriptionally activated via the NF-κB pathway in HTLV-I transformed cells [19]. Thus, based on the evidence provided by previous studies, we speculated that As_2_O_3_-induced apoptosis involves regulation of components in signal transduction pathways, such as the hTERT and NF-κB pathways, in leukemia cells. In the present study, we examined the effect of As_2_O_3_ on the survival and apoptosis of MUTZ-1 and SKM-1 cells, two MDS derived cell lines [20], [21]. In addition, we determined a possible molecular mechanism of As_2_O_3_-induced apoptosis by evaluating the expression levels of hTERT and NF-κB in MDS undergoing apoptosis.

## Materials and Methods

### Cell Culture

The MUTZ-1 cell line was established from a 5-year-old girl with MDS (FAB subtype refractory anemia with an excess of blasts), and was kindly provided by Dr. ZB Hu (University of Rochester, Rochester, NY, USA) [20]. The SKM-1 cell line (JCRB0118; Japanese Collection of Research Bioresources Cell Bank, Osaka, Japan) was established from leukemic cells of a 76-year-old Japanese male patient with monoblastic leukemia following myelodysplastic syndrome [21]. The cells were cultured in RPMI 1640 medium supplemented with 2 mM L-glutamine, 10% fetal calf serum, 50 IU/ml penicillin, and 50 µg/ml streptomycin at 37°C with 5% CO_2_ in air.

### Reagents and Treatment of MUTZ-1 and SKM-1 Cells

As_2_O_3_ was from Yida Pharmaceuticals Ltd. (Shandong, China), pyrrolidine dithiocarbamate (PDTC) from Sigma (Sigma-Aldrich, St. Louis, MO, USA), and caspase-8 inhibitor was from AnaSpec (Fremont, CA, USA). For each experiment, the reagents were freshly prepared in phosphate buffered saline (PBS) and used at the final concentrations specified in the figure legends. Untreated cells cultured in RPMI 1640 medium were used as controls. The cells were incubated with the reagents for various amounts of time at 37°C, and then the reagents were removed by washing the cells with RPMI 1640 medium. The cells were subsequently harvested for further experiments as designated.

### Determination of Cell Proliferation

An MTT [3-(4,5-dimethylthiazol-2-yl)-2,5 diphenyltetrazolium bromide; Sigma-Aldrich] assay was used to assess the effect of As_2_O_3_ on MUTZ-1 and SKM-1 cells proliferation. Briefly, 5×10^4^ cells were seeded in 96-well plates and incubated with As_2_O_3_ for 24 h, 48 h, and 72 h. At the end of the As_2_O_3_ incubation periods, MTT reagent (20 µl; 5 mg/ml in PBS) was added to each well, and the plates were incubated for 4 h at 37°C. The supernatants were carefully removed from each well and replaced with dimethyl sulfoxide (DMSO; 200 µl/well). The optical density (OD) of dissolved formazan crystal was then measured using a spectrophotometer at a wavelength of 570 nm.

### Morphological Analysis

To evaluate the effects of As_2_O_3_ on cell morphology, MUTZ-1 and SKM-1 cells were exposed to 0, 1.0, 2.0, 4.0, and 8.0 µM As_2_O_3_ for 48 h. The cells were then harvested and spun on to slides. Cytospin slides (ThermoFisher Scientific, Waltham, MA, USA) were stained by Wright-Giemsa stains. Morphological examination of the cells on the slides was performed with a light microscope at 100× magnification.

### DNA Fragmentation Assay

Cells undergoing apoptosis were utilized to detect DNA fragmentation as follows: 1×10^6^ cells were collected and incubated at 37°C overnight in lysis buffer containing 50 mM Tris-HCl, pH 8.0, 5 mM ethylenediaminetetraacetic acid, 1.2% sodium dodecyl sulfate (SDS), 150 mM NaCl, and 0.2 mg/ml proteinase K; genomic DNA (gDNA) was further isolated by phenol/chloroform extraction according to the standard protocol. The gDNA was resuspended in Tris-EDTA (TE) buffer, and 10 µg of gDNA from each sample was analyzed by gel electrophoresis (60 V for ∼2 h) using 2.0% agarose gels containing ethidium bromide. The fragmented DNA in the gel was photographed with a UV transilluminator.

### Detection of Apoptotic Cells

To detect apoptotic cells after A_2_O_3_ treatment, 1×10^6^ cells were washed with cold PBS, resuspended in binding buffer (10 mM HEPES/NaOH, pH 7.4, 140 mM NaCl, 2.5 mM CaCl_2_) containing 5 µl annexin V-fluorescein isothiocyanate (FITC; PharMingen, San Diego, CA, USA) and 10 µl propidium iodide (PI; PharMingen), gently vortexed, and incubated in the dark for 15 min. Following the incubation, binding buffer (400 µl) was added to each reaction. The cells were analyzed for PI-annexin V staining using a FACS Caliber flow cytometer and Cell Quest software (BD Bioscience, San Jose, CA, USA). A total of 1×10^3^ cells were analyzed for each sample.

### Detection of hTERT mRNA by Quantitative RT-PCR

Tri-Reagent (Promega, Madison, WI, USA) was used to isolate total cellular RNA from MUTZ-1 cells according to the manufacturer's instructions. One microliter of total cellular RNA from each sample was used for synthesis of first-strand cDNA using the SuperScript first-strand synthesis system (Invitrogen) according to the manufacturer's instructions. All PCR reactions were carried out using an ABI Prism7700 Sequence Detection System (Applied Biosystems, Foster City, USA). The following primers and probes labeled with 5′-FAM and 3′-TAMRA were used to amplify hTERT (forward primer: CATTTCATCAGCAAGTTTGGAAG; reverse primer: TTTCAGGATGGAGTAGCAGAGG).

### Western Blot Analysis

To isolate cellular protein, cells were pelleted by centrifugation (200 rpm for 5 min at room temperature), washed with PBS, and resuspended in lysis buffer (50 mM Tris-HCl, pH 6.8, 140 mM NaCl, 1 mM EDTA, 1 mM EGTA, 1 mM Na_3_VO_4_, 0.1% SDS, 0.5% NP-40, and 0.2 mM PMSF). Cell lysates were collected after centrifugation at 13,000 rpm for 15 min at 4°C, and the protein concentration of each sample was determined by the Bradford method. Equal amounts of cell lysate (50 µg of total protein) were loaded onto 10% or 12.5% SDS-polyacrylamide gels, subjected to electrophoresis, and transferred to polyvinylidene fluoride membranes (Immobilon-P; Millipore, Billerica, MA, USA). The membranes were incubated with antibodies against the following proteins: caspase-3 (Santa Cruz Biotechnology, USA), caspase-8 (Cell Signaling Technology, USA), BCL-XL (Abcam, UK), FLIP (Abcam, UK), XIAP (Abcam, UK), BCL-2 (Bioworld, USA) and PARP (Cell Signaling Technology, USA) at dilutions specified by the manufacturers. The membranes were then incubated with a secondary antibody conjugated with horseradish peroxidase and were visualized by enhanced chemiluminescence (ECL, Santa Cruz Biotechnology).

### Nuclear Protein Extraction and Gel Shift Assay

Nuclear extracts were isolated from either untreated or As_2_O_3_ treated cells. Cells (1×10^7^) were harvested, washed with cold PBS twice, and resuspended in 400 µl hypotonic buffer A (10 mM KCl, 10 mM Hepes, pH 7.9, 0.1 mM EDTA, 0.1 mM EGTA, 1.5 mM MgCl_2_, 1 mM DTT, 0.5 mM PMSF, 10 µg/ml aprotinin, and 10 µg/ml leupeptin) for 15 min on ice. The cells were lysed by adding 25 µl 10% NP-40. After centrifugation for 30 s at 13,000 rpm, the nuclear pellets were resuspended in 50 µl of lysis buffer B (25% glycerol, 20 mM Hepes, pH 7.9, 400 mM NaCl, 1 mM EDTA, 1 mM EGTA, 1 mM DTT, and 1 mM PMSF) for 30 min on ice. Nuclear extracts were collected after centrifugation at 13,000 rpm for 15 min. The Bradford method was used to determine the protein concentrations of the extracts. Electrophoretic mobility shift assays (EMSAs) were performed according to the manufacturer's protocol. The gel shift assay system was purchased from Promega (E3300), and [γ-^32^P] ATP was purchased from Furui Life Sciences (Beijing, China). T4 kinase was used to label double-stranded NF-κB consensus oligonucleotide with [γ^32^P] ATP. To conduct the DNA binding assays, 5 µg of nuclear extract was preincubated with 2 µl of binding buffer [20% glycerol, 5 mM MgCl_2_, 2.0 mM EDTA, 2.5 mM DTT, 250 mM NaCl, 50 mM Tris-HCl, and 0.25 mg/ml poly (dI): poly (dC)] in an 8 µl reaction volume for 10 min at room temperature. Next, 2 µl of labeled oligo (1 ng) was added to the reaction, and the samples were incubated for an additional 20 min at room temperature. The DNA/protein complexes were electrophoresed on 4% non-denaturing polyacrylamide gels. The gels were then dried and processed for autoradiography. Oligonucleotide Sequence: NF-κB: 5′-AGT TGA GGG GAC TTT CCC AGG C-3′ and 3′-TCA ACT CCC CTG AAA GGG TCC G-5′, SP1: 5′-ATT CGA TCG GGG CGG GGC GAG C-3′ and 3′-TAA GCT AGC CCC GCC CCG CTC G-5′, AP1: 5′-CGC TTG ATG AGT CAG CCG GAA-3′ and 3′-GCG AAC TAC TCA GTC GGC CTT-5′.

## Results

### As_2_O_3_ Inhibits the Proliferation of MUTZ-1 and SKM-1 Cells

**Figure 1:**
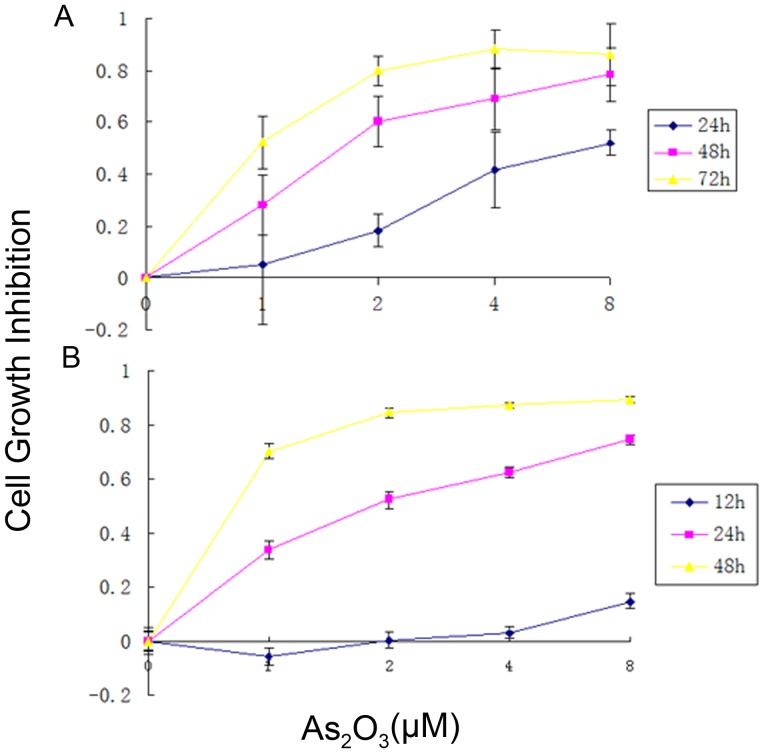
*In vitro* inhibitory effect of As_2_O_3_ on MUTZ-1 cells and SKM-1 cells proliferation. (**A**): MUTZ-1 cells were incubated with the indicated concentrations of As_2_O_3_ for 24 h, 48 h, and 72 h. (**B**): SKM-1 cells were incubated with the indicated concentrations of As_2_O_3_ for 12 h, 24 h, and 48 h. The MTT assay was performed in triplicate. The results are given as the means ± standard deviation (SD) of three independent experiments.

First we evaluated the effect of As_2_O_3_ on MUTZ-1 and SKM-1 cells proliferation using the MTT assay. Figure 1 demonstrates that As_2_O_3_ treatment produced dose- and time-dependent inhibitory effects on MUTZ-1 and SKM-1 cells proliferation. At 1.0–8.0 µM, As_2_O_3_ inhibited MUTZ-1 cell proliferation in a dose-dependent manner ranging from approximately 5% inhibition at 1 µM to 52% inhibition at 8 µM after 24 h exposure. Proliferation of the cells was inhibited by 28–78% and 52–89% after exposure to As_2_O_3_ for 48 h and 72 h, respectively (Figure 1A). Based on the data generated by the MTT assay, the IC_50_ of As_2_O_3_ for MUTZ-1 cells was 6.32 µM for a 24 h exposure, 1.94 µM for a 48 h exposure, and 0.59 µM for a 72 h exposure. It was approximately -5% to 14% inhibition after 12 h exposure to As_2_O_3_, 33% to 74% after 24 h and 70–89% after 48 h in SKM-1 cells (Figure 1B). The SKM-1 cells were more sensitive to As_2_O_3_ than the MUTZ-1 cells. The maximum inhibition of As_2_O_3_ treatment to SKM-1 cells was at 48 h while it was 72 h in MUTZ-1 cells.

### As_2_O_3_ Induces MUTZ-1 and SKM-1 Cells Apoptosis

**Figure 2:**
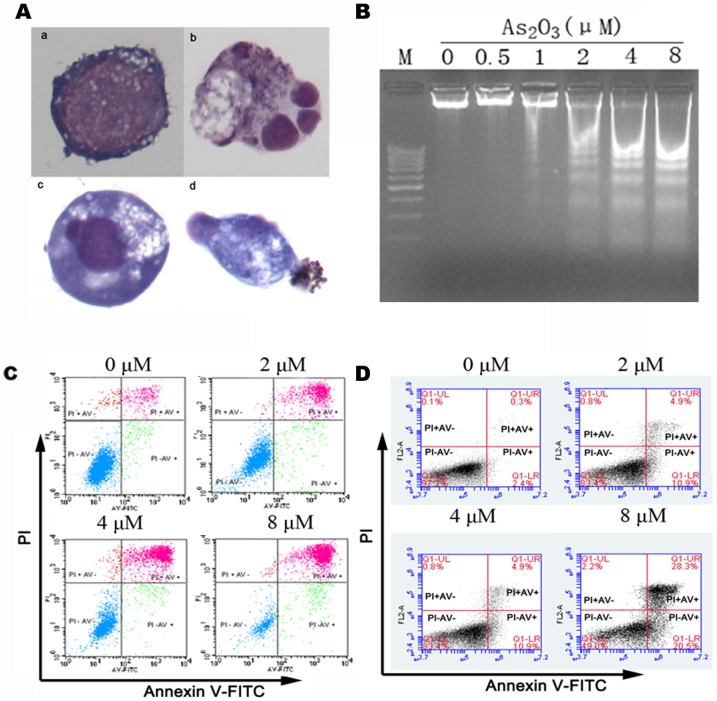
Detection of MUTZ-1 and SKM-1 cells apoptosis after treatment with As_2_O_3_. (**A**): Morphologic evaluation of apoptotic cells using Wright-Giemsa staining. (a) untreated cells; (b) apoptotic cells with nuclear karyorrhexis; (c) apoptotic cells with nuclear pyknosis; (d) apoptotic cell with apoptotic bodies. Magnification is 100×, using an Olympus microscope. (**B**): Detection of DNA ladder after 24 h of As_2_O_3_ treatment. Cellular DNA was isolated and separated by 2% agarose gel electrophoresis. M = 100 bp DNA marker. (**C**) and (**D**): Flow cytometry analysis of cell apoptosis. After treatment with As_2_O_3_, the cells were double stained with annexin V-FITC and PI and analyzed by flow cytometry. Annexin V positive cells are shown in the second and fourth quadrants of the plots. (**C**): Percent of apoptotic MUTZ-1 cells were 5.89%±1.56%, 29.62%±9.95%, 43.88%±15.08%, and 74.8%±7.97% after 24 h treatment of the cells with 0, 2.0, 4.0, and 8.0 µM of As_2_O_3_, respectively. (**D**): Percent of apoptotic SKM-1 cells were 2.6%, 15.8%, 30.8%, and 48.7% after 24 h treatment of the cells with 0, 2.0, 4.0, and 8.0 µM of As_2_O_3_, respectively. Values represent means ± standard deviation (SD) of three individual experiments.

Next, we tested whether As_2_O_3_ treatment inhibited MUTZ-1 and SKM-1 cells proliferation through the induction of apoptosis. Evaluation of the morphology of MUTZ-1 and SKM-1 cells treated with As_2_O_3_ revealed that at concentrations of 1.0–8.0 µM, MUTZ-1 and SKM-1 cells displayed significant nuclear condensation and fragmentation as well as apoptotic bodies, typical of cell apoptosis (Figure 2A). DNA fragmentation in As_2_O_3_ treated MUTZ-1 and SKM-1 cells were detected by agarose gel electrophoresis (Figure 2B). Apoptosis in As_2_O_3_-treated MUTZ-1 and SKM-1 cells were also confirmed by staining with annexin V-FITC and PI and subsequent analyses by flow cytometry. As seen in Figure 2C and Figure 2D, the proportion of apoptotic cells increased progressively over 24 h, corresponding to the increased dose of As_2_O_3_. These data suggest that As_2_O_3_ induces MUTZ-1 and SKM-1 cells apoptosis and that this may be one mechanism by which MUTZ-1 and SKM-1 cells proliferation are inhibited by As_2_O_3_.

### As_2_O_3_ Induces Caspase-3 and Caspase-8 Activity and PARP Cleavage

**Figure 3:**
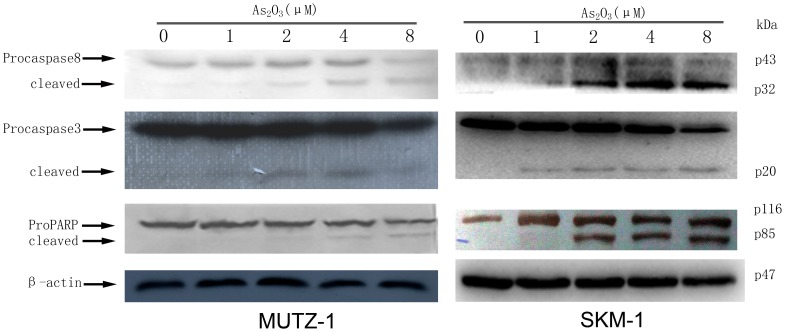
Cleavage of caspase-8, caspase-3, and poly (ADP-ribose) polymerase (PARP) in MUTZ-1 and SKM-1 cells treated with As_2_O_3_ for 24 h. The concentrations of As_2_O_3_ used to treat MUTZ-1 and SKM-1 cells in the experiments are indicated on top of the figure. To control for equal loading, anti-β-actin and GAPDH antibody were used as probe in MUTZ-1and SKM-1 cells respectively.

Caspases regulate apoptosis. For example, caspase-3 has been demonstrated to induce apoptosis in response to many different stimuli. After treatment of MUTZ-1 and SKM-1 cells with As_2_O_3_ at various concentrations (1.0–8.0 µM) for 24 h, we detected activation of caspase-3 and caspase-8 in a dose-dependent manner. As shown via Western blot in Figure 3, we detected the lower molecular weight cleavage products of caspase-3 and caspase-8, including the 20 kDa and 42 kDa active forms of the proteins. To further evaluate the role of caspases in As_2_O_3_-induced apoptosis, we evaluated PARP cleavage because PARP is a general caspase substrate in cells undergoing apoptosis. As shown in Figure 3, Western blotting revealed that both the uncleaved proform of PARP (116 kDa) and the cleavage fragment of PARP (89 kDa) were observed in MUTZ-1 and SKM-1 cells treated with As_2_O_3_ (1.0–8.0 µM) for 24 h.

### As_2_O_3_ Induces MUTZ-1 and SKM-1 cells apoptosis were BCL-2, BCL-XL and FLIP independent

**Figure 4:**
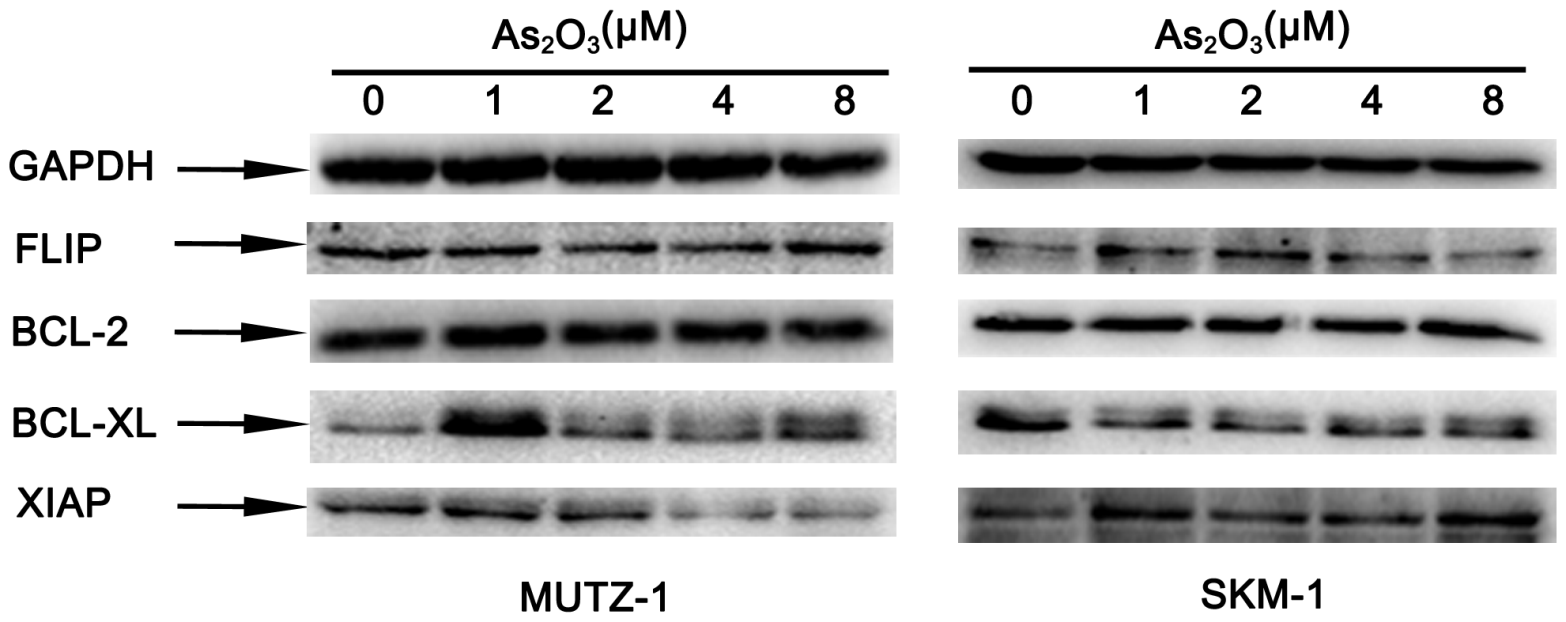
Expression of FLIP, BCL-2, BCL-XL, XIAP in MUTZ-1 and SKM-1 cells treated with As_2_O_3_ for 24 h. The concentrations of As_2_O_3_ used to treat MUTZ-1 and SKM-1 cells in the experiments are indicated on top of the figure. To control for equal loading, a GAPDH antibody was used as a probe.

BCL-2, BCL-XL, FLIP and XIAP are important apoptosis related proteins. After treatment of MUTZ-1 and SKM-1 cells with As_2_O_3_ at various concentrations (1.0–8.0 µM) for 24 h, we detected expressions of BCL-2, BCL-XL, XIAP, and FLIP proteins via Western blot. As shown in Figure 4, expressions of BCL-2, BCL-XL and FLIP had no changes in MUTZ-1 and SKM-1 cells after As_2_O_3_ treatment at various concentrations. XIAP expression was down regulated in MUTZ-1 cells, but it was not seen in SKM-1 cells.

### As_2_O_3_ Downregulates hTERT mRNA and Protein Expression in MUTZ-1and SKM-1 Cells

**Figure 5:**
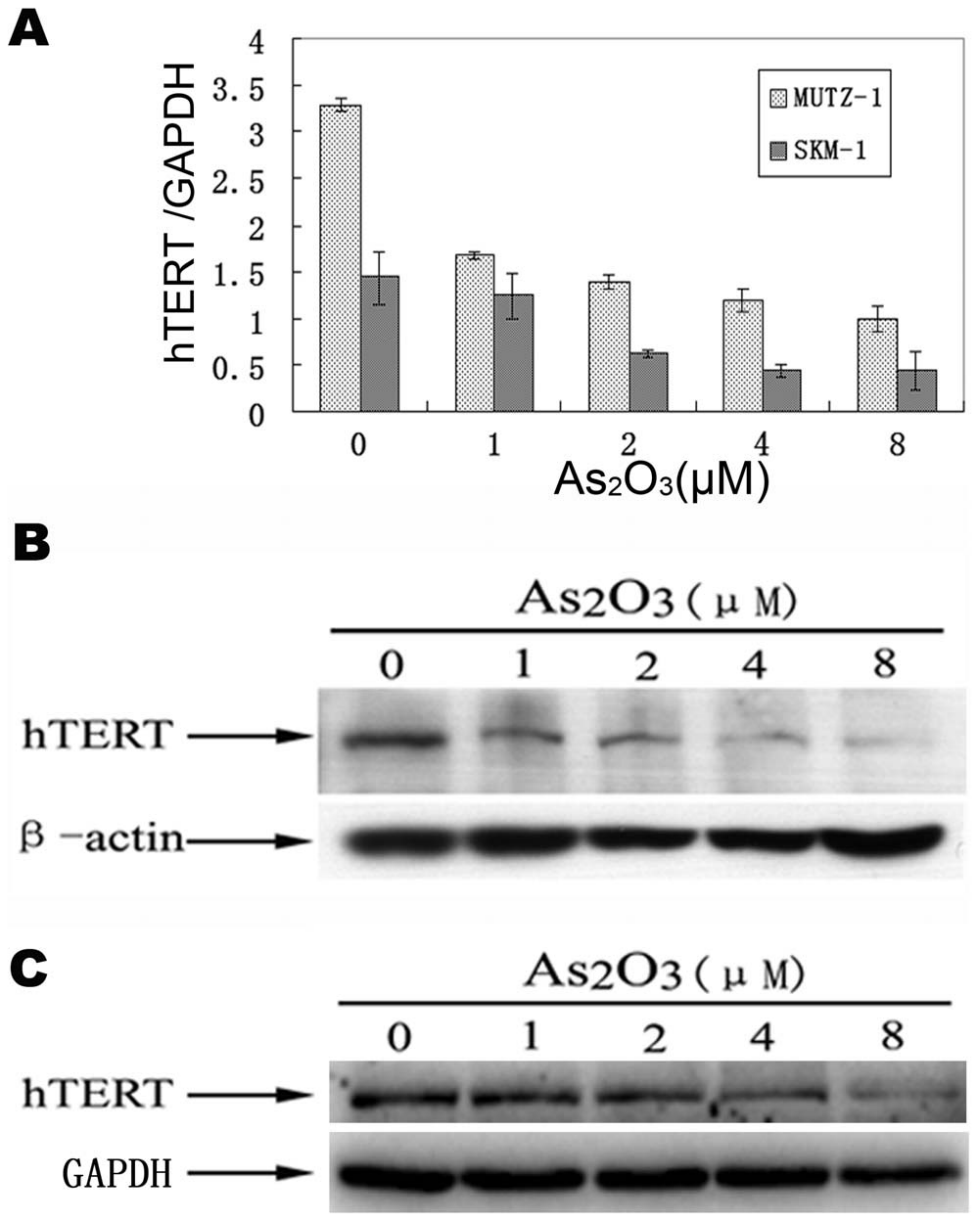
Expressions of hTERT mRNA and protein of MUTZ-1 and SKM-1 cells evaluated by Quantitative RT-PCR and Western blotting. (**A**): Expressions of hTERT mRNA of MUTZ-1 and SKM-1 cells evaluated by Quantitative RT-PCR. (**B**): Expression of hTERT protein in MUTZ-1 cells treated with As_2_O_3_ evaluated by Western blot. (**C**): Expression of hTERT protein in SKM-1 cells treated with As_2_O_3_ evaluated by Western blot. The concentrations of As_2_O_3_ (µM) are indicated on the tops of the figures. To control for equal loading, anti-β-actin and GAPDH antibody were used as probe. Results are representative of three independent experiments.

It is known that hTERT participated in tumorigenesis via elongation of the telomere, increasing anti-apoptotic capacity, enhancing DNA repair, maintaining stem cells and regulating gene expression, etc. Therefore, hTERT has important roles in cell survival and tumorigenesis. To elucidate whether hTERT participates in As_2_O_3_-induced MUTZ-1 and SKM-1 cells apoptosis, we utilized Quantitative RT-PCR to examine hTERT expression levels in MUTZ-1 and SKM-1 cells treated with various concentrations of As_2_O_3_ for 24 h. As shown in Figure 5A, As_2_O_3_ treatment significantly downregulated the expression of hTERT mRNA in a dose-dependent manner. Meanwhile, we detected hTERT protein in MUTZ-1 and SKM-1 cells treated with various concentrations of As_2_O_3_ for 24 h via Western blot. As shown in Figure 5B and 5C, As_2_O_3_ treatment significantly downregulated the expression of hTERT protein in a dose-dependent manner.

### As_2_O_3_ Inhibits MUTZ-1 and SKM-1 cells NF-κB DNA Binding

**Figure 6:**
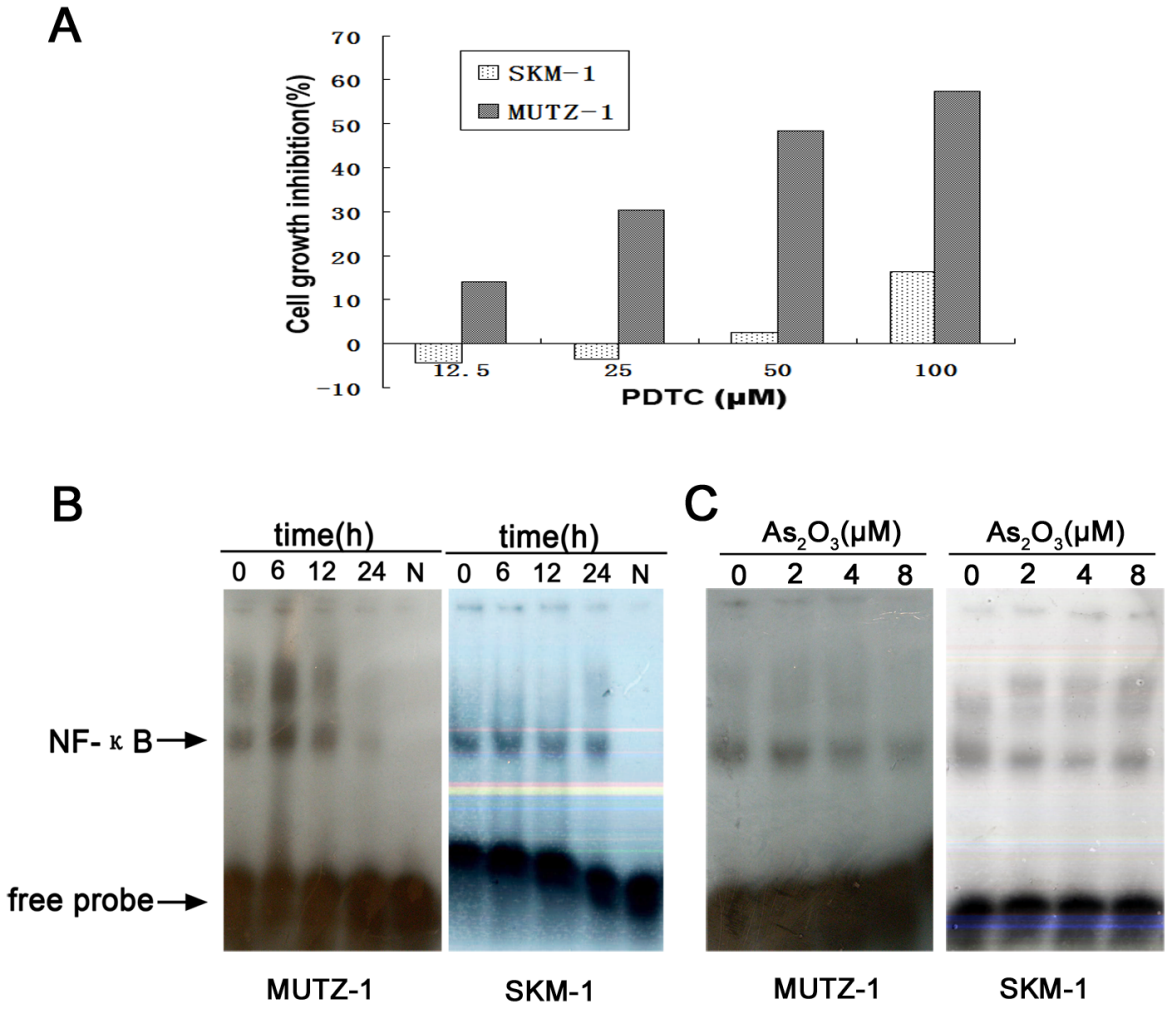
Effect of PDTC on MUTZ-1 and SKM-1 cells proliferation and Gel shift assay of NF-κB in As_2_O_3_-treated MUTZ-1 and SKM-1 cells. (**A**) Effect of PDTC on MUTZ-1 and SKM-1 cells proliferation. MUTZ-1 and SKM-1 cells were treated for 10 h with indicated concentrations of PDTC. The experiments were performed in triplicate and MTT assays were used to evaluate proliferation. Data are given as means ± standard deviation (SD) of three independent experiments. (**B**) and (**C**) Represents time and dose responses of NF-κB in MUTZ-1 and SKM-1 cells treated with different concentration of As_2_O_3_. (N =  negative control).

NF-κB protects cells against apoptosis and is a transcriptional regulator of hTERT. Therefore, we investigated whether As_2_O_3_ inhibited NF-κB expression in MUTZ-1 and SKM-1 cells. PDTC is an antioxidant and an inhibitor of NF-κB. First, MTUZ-1 and SKM-1 cells were treated with PDTC in order to identify possible role of NF-κB in proliferation of MUTZ-1 and SKM-1 cells. As shown in Figure 6A, MTUZ-1 growth was significantly inhibited after incubation with PDTC at 12.5 to 100 µM concentrations. It demanded a high concentration of PDTC to inhibit SKM-1 cells growth. As shown in figure 6A, 100 µM PDTC inhibited SKM-1 cells growth significantly, but 12.5 to 50 µM PDTC had no effect on SKM-1 cells growth. Then, we performed gel shift assays using nuclear extracts from As_2_O_3_-treated MUTZ-1 and SKM-1 cells to determine the DNA binding activities of NF-κB. As shown in Figure 6B and 6C, incubating MUTZ-1 and SKM-1 cells with As_2_O_3_ significantly inhibited NF-κB DNA binding activity. These inhibitory effects were time-dependent (Figure 6B) and dose-dependent (Figure 6C). Compared with MUTZ-1 cells, NF-κB was downregulated mildly in SKM-1 cells.

### As_2_O_3_ Inhibits MUTZ-1 cells SP-1 and AP-1 DNA Binding

**Figure 7:**
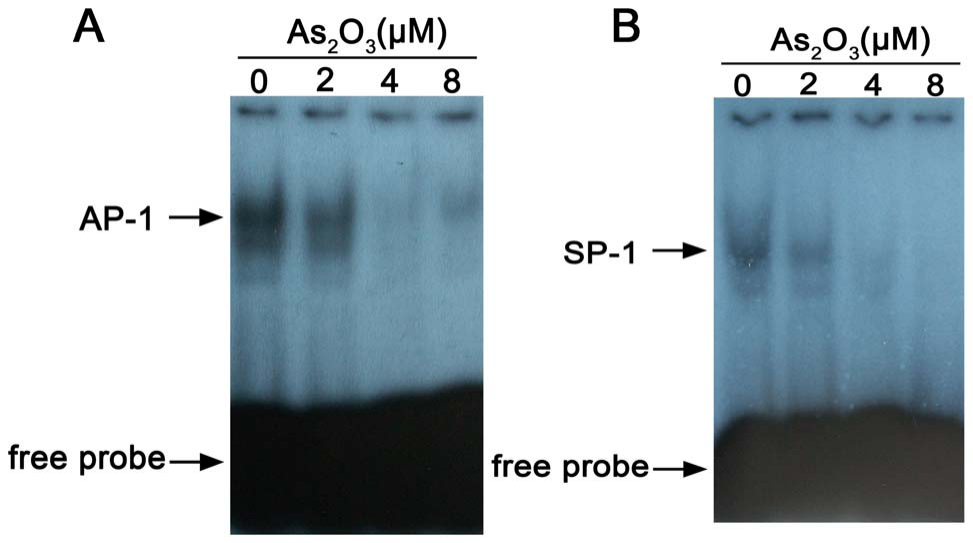
Gel shift assay of AP-1 and SP-1 in As_2_O_3_-treated MUTZ-1. Represents dose responses of AP-1 and SP-1 in MUTZ-1 cells treated with different concentration of As_2_O_3_. Results are representative of three independent experiments.

Furthermore, the DNA binding activities of SP-1 and AP-1, known transcriptional regulators of hTERT, were also found to be significantly inhibited in MUTZ-1 cells by As_2_O_3_ treatment, as demonstrated in Figure 7A and Figure 7B.

### As_2_O_3_-Induced Reduction in NF-κB Activity and hTERT expression of MUTZ-1 is Caspase-8 Independent

**Figure 8:**
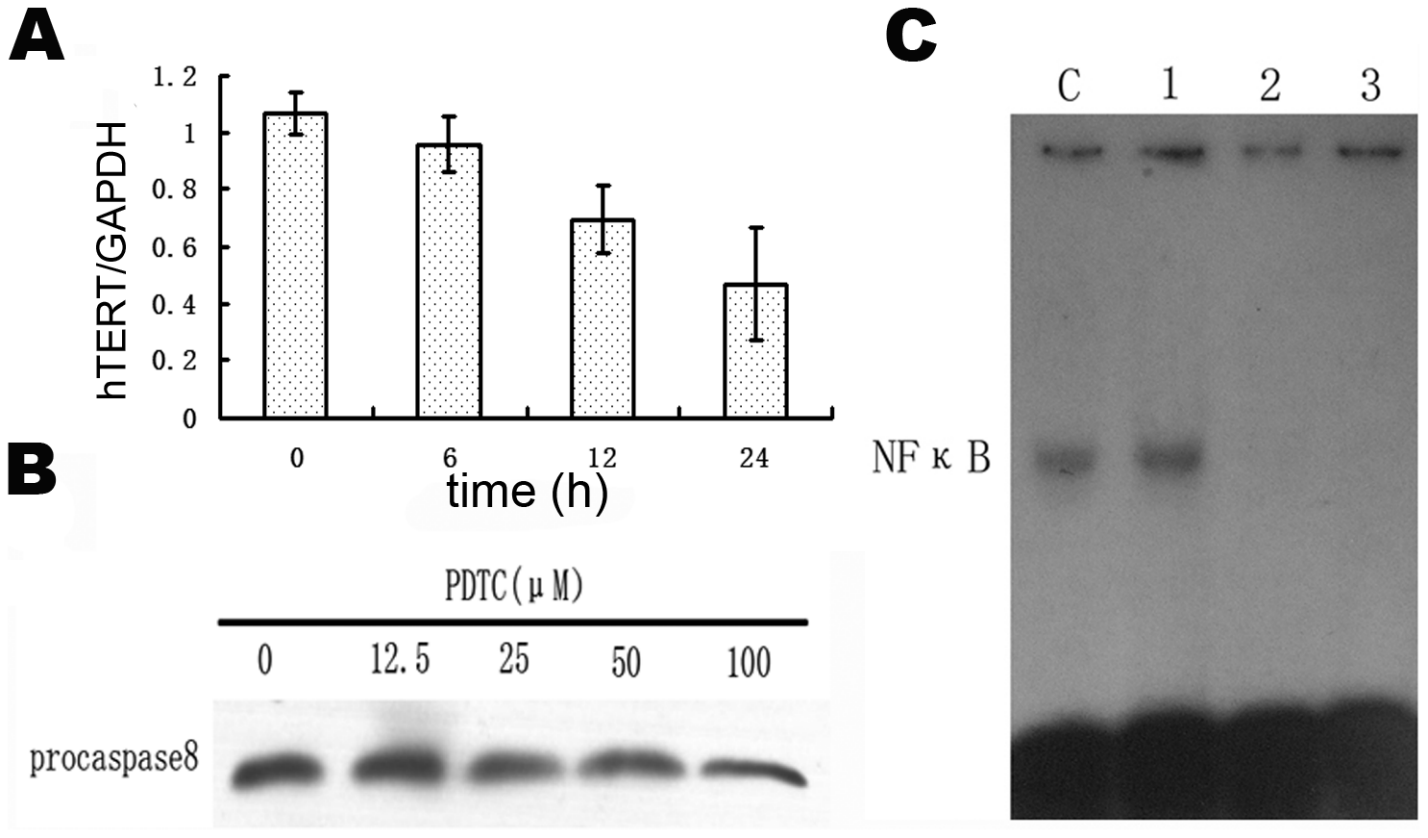
Effects of PDTC and caspase-8 inhibitor on MUTZ-1 cells. (**A**) Effects of PDTC on MUTZ-1 cell hTERT mRNA expression. Detection of hTERT mRNA from MUTZ-1 cells treated with 100 µM PDTC for the indicated times using Quantitative RT-PCR. (**B**) Effects of PDTC on MUTZ-1 cell caspase-8 protein level. Western blot analysis of caspase-8 protein from MUTZ-1 cells treated with different concentrations of PDTC for 10 h. The doses of PDTC used are expressed in µM. (**C**) Effects of caspase-8 inhibitor and As_2_O_3_ on NF-κB activity as evaluated by gel shift assays. C =  control; lane 1 =  treatment with 40 µM caspase-8 inhibitor; lane 2 =  treatment with 8.0 µM As_2_O_3_; lane 3 =  treatment with 40 µM caspase-8 inhibitor plus 8.0 µM As_2_O_3_. Marked inhibition of NF-κB activity by As_2_O_3_ alone, and by As_2_O_3_ plus caspase-8 inhibitor was observed, while caspase-8 inhibitor alone showed little effect on NF-κB activity when compared with controls (lane C, and lane 1).

To elucidate the potential relationship between caspase-8, NF-κB and hTERT, in the As_2_O_3_-induced apoptosis of MUTZ-1 cells, the expression of hTERT mRNA were evaluated by Quantitive RT-PCR in MUTZ-1 cells treated with 100 µM PDTC for 6 h to 24 h firstly. As shown in Figure 8A, hTERT mRNA expression was downregulated. Meanwhile Western blotting could not detect the specific cleavage of caspase-8 in MUTZ-1 cells treated with PDTC for 10 h (Figure 8B). Then MUTZ-1 cells were treated with 40 µM caspase-8 inhibitor either alone or in combination with 8.0 µM As_2_O_3_. As shown in Figure 8C, treatment of MUTZ-1 cells with 40 µM caspase-8 inhibitor alone had no significant effect on NF-κB activity. However, As_2_O_3_ treatment of the cells either alone or in combination with caspase-8 inhibitor resulted in significant inhibition of NF-κB activity. This data suggests that caspase-8 is unlikely to participate in the As_2_O_3_-induced reduction of NF-κB activity and hTERT downregulation.

## Discussion

As_2_O_3_ has been used for many years in clinics for treating various hematological malignancies [1]–[3], [7], [8], [10], [11]. It is an effective therapeutic reagent for treating hematologic malignancy because of its pro-apoptotic, anti-proliferative, and anti-angiogenic effects. Using cell and molecular biological approaches, we investigated the effects of As_2_O_3_ on two MDS cell lines, MUTZ-1 and SKM-1 cells, and explored potential mechanisms for induction of apoptosis in these cells. Our results demonstrated that As_2_O_3_ had a strong anti-proliferative activity in MUTZ-1 and SKM-1 cells.

The MTT assay demonstrated that As_2_O_3_ strongly inhibited MUTZ-1 and SKM-1 cells proliferation in a dose- and time-dependent manner. MUTZ-1 and SKM-1 cells proliferation were significantly inhibited after 48 h to 72 h by As_2_O_3_ concentrations ranging from 0.5–2.0 µM. Our observation is consistent with previous findings that As_2_O_3_ induced apoptosis in many types of cancer cell lines at concentrations higher than 0.5 µM [13], [22]. We further demonstrated that As_2_O_3_ treatment induced MUTZ-1 and SKM-1 cells apoptosis, as demonstrated by morphological alteration, DNA fragmentation, PI and annexin V staining, and caspase-3, caspase-8, and PARP cleavage. Some other apoptosis proteins, FLIP, BCL2 and BCL-XL, didn't participate in MUTZ-1 and SKM-1 cells apoptosis induce by As_2_O_3_. Role of XIAP in apoptosis of these two cell lines induced by As_2_O_3_ in unclear because results isn't consistent in these two cell lines.

We also observed that hTERT mRNA and protein expression were suppressed in As_2_O_3_-treated MUTZ-1 and SKM-1 cells. The suppression of hTERT was possibly due to inhibition of NF-κB, a transcription factor known to regulate hTERT. This data agrees with that from previous studies showing that As_2_O_3_ promotes apoptosis by inhibiting telomerase activity and transcription of the hTERT gene. hTERT elongates the telomere by telomerase-dependent mechanisms, and decreased telomerase activity results in increased cell death or apoptosis [23], [24]. Stewart et al. [25] found that telomerase contributes to tumorigenesis by a mechanism that is independent of telomere length. Telomerase forms protective caps at the end of telomeres. These cap protect the ends of chromosomes from end-to-end fusions and illegitimate recombination events [25], [26]. Because multiple studies have shown that the reduction of telomerase transcription by As_2_O_3_ may be caused by a direct effect of arsenic on transcription factors such as Sp-1 and Myc [27], we speculated that As_2_O_3_ might regulate hTERT expression by affecting the DNA binding activities for NF-κB, SP-1, and AP-1. Gel shift assays, demonstrated that As_2_O_3_ inhibited the DNA binding activities of all the three transcription factor. These data indicate that hTERT is an important molecular target of As_2_O_3_. Thus, one possible mechanism for As_2_O_3_-induced apoptosis is the suppression of hTERT mRNA expression via the inhibition of several transcription factors, including NF-κB, SP-1, and AP-1.

NF-κB is an important transcriptional regulator of hTERT and is associated with MM cell growth, survival, and drug resistance [28]. NF-κB p65 regulates telomerase activity by modulating the hTERT [29]. Functions of NF-κB in diverse tumor cells include maintaining proliferation and protecting cells from apoptosis [30]. Our data indicated that As_2_O_3_ inhibited NF-κB activity in MUTZ-1 and SKM-1 cells, which is consistent with the finding that NF-κB inhibition contributes to arsenic-mediated induction of apoptosis as reported by Mathas et al. [15]. To determine whether inhibition of NF-κB activity reduces the expression of hTERT in MUTZ-1 cells, the cells were treated with PDTC, a specific inhibitor of NF-κB, and hTERT mRNA levels were measured by Quantitative RT-PCR. Our data showed that growth of MUTZ-1 cells was inhibited by 100 µM PDTC, and expression of hTERT mRNA in the cells was significantly decreased, further suggesting that As_2_O_3_ decreased hTERT mRNA through inhibition of NF-κB activity.

Because caspase activity has an important role in apoptosis, we determined if inhibition of caspase-8 activation could protect cells from apoptosis. Specific cleavage of caspase-8 in MUTZ-1 cells treated by PDTC was detected by Western blot analysis. Our data indicated that PDTC had no effect on caspase-8 activation, although a strong inhibition of MUTZ-1 cell growth by PDTC was observed. Incubation of MUTZ-1 cells with caspase-8 inhibitor failed to prevent the inhibition of NF-κB activity by As_2_O_3_, suggesting that As_2_O_3_-induced apoptosis occurs via two independent pathways in MUTZ-1 cells: first, by activation of caspase-8; and second, by inhibition of NF-κB activity, which subsequently results in downregulation of hTERT expression.

Some contradictory reports on the effects of As_2_O_3_ on MDS cells have been reported. Sanz et al. [31] analyzed NF-κB activity in 17 bone marrow samples from MDS patients and found that six of them showed a significant increase in NF-κB activity as assessed by ELISA. Increased NF-κB activity was found mainly in MDS patients with refractory anemia (4 of 5 cases), in which apoptosis was increased rather than decreased. They further reported that NF-κB might increase apoptosis in bone marrow cells by upregulating NF-κB dependent apoptotic cytokines, such as TNFα and FasL. Kerbauy et al. [9] found that patients with low grade/early stage MDS [refractory anemia/refractory anemia with ring sideroblasts (RA/RARS)] had low NF-κB activity levels that were comparable to those in normal marrow, while patients with RA with excess blasts (RAEB) had significantly increased NF-κB activity levels. Treatment with As_2_O_3_ (2–200 µM) inhibited NF-κB activity in primary MDS and downregulated the expression of Bcl-XL, Bcl-2, XIAP, and FLIP. The expression of these anti-apoptotic proteins is NF-κB-dependent and their downregulation led to apoptosis in primary MDS. In contrast, overexpression of FLIP increased NF-κB activity and promoted ML1 cell resistance to As_2_O_3_-induced apoptosis. In another independent study, Woo et al. [32] found that As_2_O_3_ sensitized human cervical cancer cells to CD95/Fas induced apoptosis through reactive oxygen species mediated upregulation of CD95/Fas and by NF-κB activation. Thus, we speculate that NF-κB may play either apoptotic or anti-apoptotic roles in tumorigenesis depending on its target, on the cell type, and on the form of arsenate used.

Taken together, we conclude that As_2_O_3_ induces MUTZ-1 and SKM-1 cells apoptosis by two independent pathways: 1) by activation of caspase-3, caspase-8, and PARP; and 2) by inhibition of NF-κB activity, which results in the downregulation of hTERT expression. Thus, hTERT and NF-κB are two important proteins involved in As_2_O_3_-induced apoptosis. In addition to NF-κB, As_2_O_3_ might also inhibit expression of hTERT via inhibition of the activity of transcription factors SP-1 and AP-1.
